# Dynamic Prestress in a Globular Protein

**DOI:** 10.1371/journal.pcbi.1002509

**Published:** 2012-05-10

**Authors:** Scott A. Edwards, Johannes Wagner, Frauke Gräter

**Affiliations:** 1 CAS-MPG Partner Institute and Key Laboratory for Computational Biology, Shanghai, China; 2 Heidelberg Institute for Theoretical Studies, Heidelberg, Germany; National Cancer Institute, United States of America and Tel Aviv University, Israel, United States of America

## Abstract

A protein at equilibrium is commonly thought of as a fully relaxed structure, with the intra-molecular interactions showing fluctuations around their energy minimum. In contrast, here we find direct evidence for a protein as a molecular tensegrity structure, comprising a balance of tensed and compressed interactions, a concept that has been put forward for macroscopic structures. We quantified the distribution of inter-residue prestress in ubiquitin and immunoglobulin from all-atom molecular dynamics simulations. The network of highly fluctuating yet significant inter-residue forces in proteins is a consequence of the intrinsic frustration of a protein when sampling its rugged energy landscape. In beta sheets, this balance of forces is found to compress the intra-strand hydrogen bonds. We estimate that the observed magnitude of this pre-compression is enough to induce significant changes in the hydrogen bond lifetimes; thus, prestress, which can be as high as a few 100 pN, can be considered a key factor in determining the unfolding kinetics and pathway of proteins under force. Strong pre-tension in certain salt bridges on the other hand is connected to the thermodynamic stability of ubiquitin. Effective force profiles between some side-chains reveal the signature of multiple, distinct conformational states, and such static disorder could be one factor explaining the growing body of experiments revealing non-exponential unfolding kinetics of proteins. The design of prestress distributions in engineering proteins promises to be a new tool for tailoring the mechanical properties of made-to-order nanomaterials.

## Author Summary

A tensegrity structure is one composed of members that are permanently under either tension or compression, and the balance of these tensile and compressive forces provides the structure with its mechanical stability. Macroscale tensegrity structures, which include Buckminster Fuller's geodesic domes, achieve exceptional structural integrity with a minimal use of resources. The question we address in this work is whether nature makes use of molecular-scale tensegrity in the design of proteins. Using Molecular Dynamics simulations of the protein ubiquitin, we measure the network of pairwise forces connecting the amino acid residues and show that this network does indeed have the character of a tensegrity structure. Furthermore, we find that the arrangement of tensile and compressive forces is such that hydrogen bonds in the protein's beta sheet, which are crucial for bearing mechanical loads, are compressed. This pre-compression is enough to significantly lengthen the lifetime of a bond under a given force, and thus should be an important factor in determining the protein's mechanical strength. The rational design of molecular prestress networks promises to be a new avenue for the engineering of proteins with made-to-order mechanical properties, for applications in medicine, materials and nanotechnology.

## Introduction

The principle of ‘minimal frustration’ [1], [2] underlies the thermodynamic picture of protein folding. According to this picture, proteins negotiate a rough, funnel-shaped energy landscape during the folding process, and eventually settle in a state that, as much as possible, satisfies the energetic constraints arising from the multitude of interatomic covalent, electrostatic and van der Waals interactions. Although frustration is minimised in the native state, it is not completely eradicated. Even in the simplest crystals, the equilibrium state is one that minimises the energy of the structure as a whole, not every atom-atom interaction individually; global constraints prevent every pairwise interactions from being perfectly satisfied. This is even more the case for proteins, in which the topological contraints of the backbone peptide bonds further restrict the freedom of individual atoms to individually satisfy every interaction.

Such local frustration in a protein must give rise to residual mechanical forces – thus, proteins are in some sense prestressed materials. D. Ingber has proposed that proteins and other biological structures should be understood in light of the architectural concept of *tensegrity* [3], [4], popularised by Buckminster Fuller, describing structures the mechanical stability of which arises purely from a balance between pre-tensed and pre-compressed members. The concept of biomolecular tensegrity has come under focus very recently in the work of Shih, Ingber, and co-workers, who have designed and synthesised prestressed DNA structures [5]; it has also been invoked in a novel method for interpreting free energy profiles inferred from the forced unfolding of single biomolecules [6]. In contrast to this tensegrity picture, classic coarse-grained models of proteins, which have been used extensively to study protein folding and dynamics, typically neglect prestress. Both G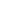-style models [7]–[9] and elastic network models [10], [11] define the equilibrium separation of every residue pair to be precisely the separation measured in the native state, and thus every residue-residue interaction is individually relaxed; as such, the native state is defined to contain no residual force. Thus, especially in research areas that rely on these coarse-grained approaches, the consequences of prestress for folding and dynamics have not been well explored.

It has been demonstrated recently [12] that, in graphene sheets, the prestress of bonds around defects at grain boundaries is the key determining factor for toughness of the sheets. This result highlights the fact that the existence of prestress can qualitatively change the mechanical properties of a structure, and raises the question of to what extent such effects are utilised by nature to tune the mechanical stability of proteins. Since concentrations of internal force in a molecule could be used to drive conformational changes if released, via thermal fluctuations or due to interactions with other molecules, the spatial distribution of prestress in a protein may also provide important clues for understanding the mechanisms for protein-protein interactions [13], [14], protein-DNA interactions [15], and allostery; indeed, the existence of ‘tensed’ and ‘relaxed’ states in allosteric proteins has been a central concept in models of allosteric transitions since the classic early models of inter-domain allostery in hemoglobin [16]–[18]. Elastic stress also plays a central role in the more recent allosteric model of Savir and Tlusty [19]. Using short lengths of pre-tensed double-stranded DNA to stretch individual molecules, it is now possible to directly observe the role played by elastic stress in the allosteric control of protein enzymes [20], [21] and ribozyme [22].

But how is such a global elastic stress built up within a protein scaffold? We used all-atom Molecular Dynamics (MD) simulations to quantify the importance of prestress in the native state of ubiquitin. To this end, we adapted an earlier technique for measuring force distributions in mechanically perturbed proteins [23], [24] to allow the calculation of effective pairwise residue-residue force profiles. This procedure is a direct force measurement, unlike other methods based on inferring pairwise forces from fluctuations [25], [26], and does not require any *a priori* assumptions about the form of the force profile. From the effective force profiles we extracted average forces for each residue pair, thereby constructing a prestress network for the protein. We found that high residual forces exist throughout the protein, and are particularly associated with hydrogen bonds and salt bridges. The magnitude of these forces is shown to be enough to significantly influence the protein's mechanical properties, most notably its unfolding pathway. We also discover that, for some side-chains, prestress is *dynamic* – inter-residue mechancial coupling switches between a number of distinct regimes depending on side-chain conformations.

## Results

### Average residual force network

**Figure 1:**
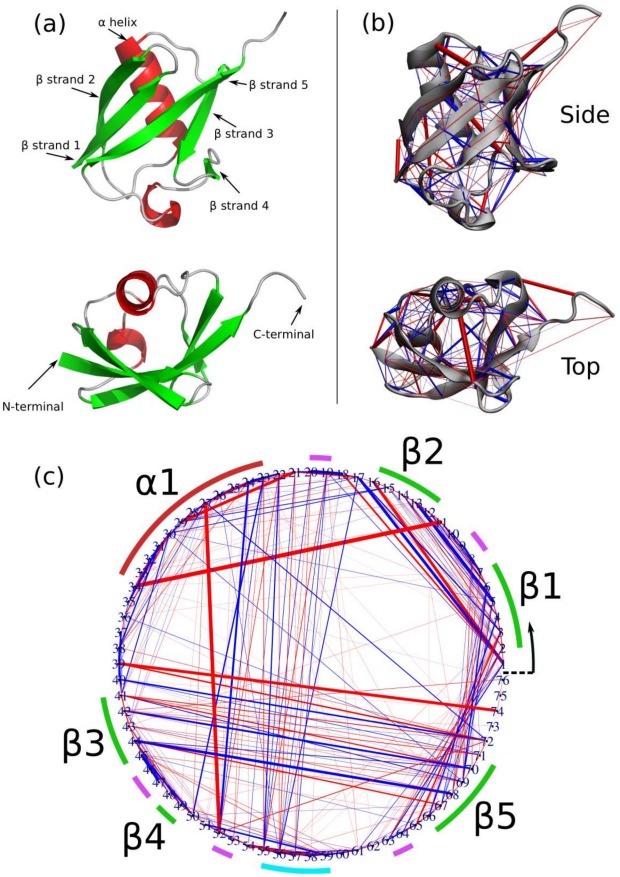
(a) X-ray structure of ubiquitin (PDB accession code 1UBI). The lower view is rotated 90 around the horizontal axis with respect to the upper view. Helices are colored red, and beta strands green. Note that the beta strands 1 and 5 are closest to the N- and C- termini respectively, and thus the interaction between them is the primary determinant of the protein's mechanical stability against stretching from the termini. (b) The network representing the inter-residue forces for ubiquitin, averaged over 100 ns of molecular dynamics simulations, superimposed on the 3D structure of the protein. The color and width of cylinders connecting residue pairs correspond to the direction and magnitude of the mean inter-residue force: blue for repulsive force, red for attractive force, and the maximum width corresponding to a force magnitude of 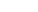 pN. (c) A circle graph representation of the prestress network in (b). Numbers around the circumference are residue indices. Colored arcs correspond to secondary structure: alpha helix (red), beta strand (green), hydrogen-bonded loop (purple) and 3–10 helix (cyan).

From 100 ns of MD trajectories, we calcuated effective force profiles for every pair of amino acid residues in ubiquitin (Fig. 1a), as described in the Methods section. The average forces inferred from these profiles are plotted in Fig. 1b superimposed on the 3D structure of the protein. For clarity, the same force network is also represented in Fig. 1c as a circular graph, with each vertex corresponding to a residue. Covalently bonded residue pairs are neglected [see the Supplementary Material (Text S1 and Figs. S6 and S7) for details on covalent bond forces]. Red (blue) edges represent attractive (repulsive) forces, and edge thicknesses correspond to the magnitude of the forces, which range between −490 pN (attractive) and +407 pN (repulsive). In the context of cell biology these are high forces – for comparison, the forces generated by the kinesin walk have been measured to be on the order of 2 pN [27]. An animation showing the projection of this network on the three-dimensional structure of the protein is provided in the Supplementary Material (Video S1).

The most obvious large-scale structures in the network are the relatively ordered bands of both tensile and compressive forces that connect neighboring beta strands: specifically, the two parallel pairs of beta strands 1/2 and 3/5, and the anti-parallel beta strand pair 1/5. In contrast, isolated cases of strongly tensed (red) residue pairs are also observed, such as Lys27-Asp52 and Lys11-Glu34, which do not correlate with neighboring residues. These high tensile forces occur only between residues with charged side-chains; as discussed in more detail below, they correspond to tensed salt bridges.

### Hydrogen bonds are pre-compressed in beta sheets, and pre-tensed in alpha helices

**Figure 2:**
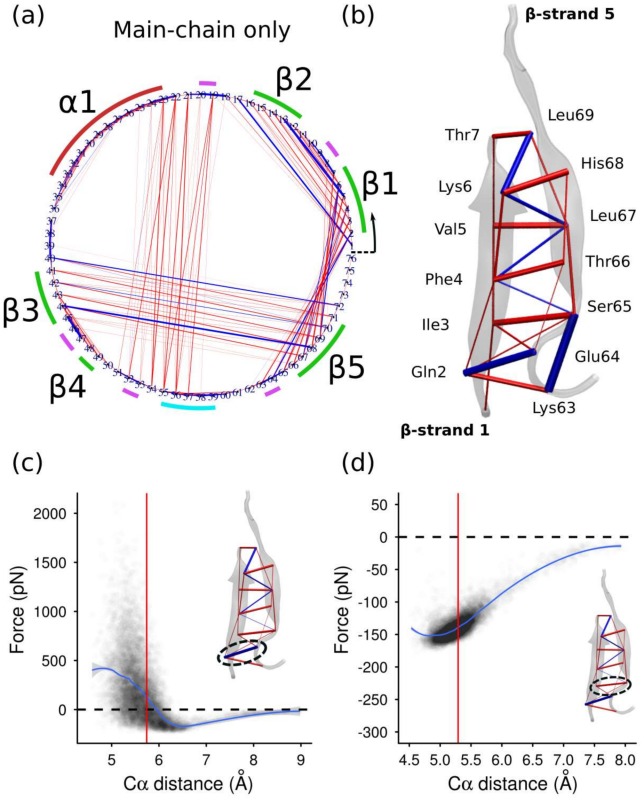
(a) As for Fig. 1c, but with only main-chain atoms (N, O, C, C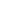, H) included in the force calculation. (b) Mean inter-residue forces (main-chain only) for beta strands 1 and 5. (c) & (d) Distributions of main-chain–main-chain force vs C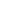 separation for the residue pairs Gln2-Glu64 (c) and Ile3-Ser65 (c). Each dot corresponds to a single frame from the MD trajectory. The vertical red line shows the mean separation, and the blue curve is a local polynomial fit to the data using the Loess method. The inset highlights the relevant residue pair in the beta sheet structure.

To get a clearer picture of the prestress pattern associated with the main-chain interactions in beta sheets, the force network accounting for only inter–main-chain interactions is shown in Fig. 2a and Video S2 [here ‘main-chain’ refers to the N, C, C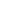, O, and H atoms making up the backbone]. Inter–main-chain interactions are found to be predominantly attractive, with a few strongly repulsive pairs. To better understand this phenomenon we examine in more detail the residue-wise force distribution in beta strands 1 and 5. These strands are of special relevance to the mechanical stability of ubiquitin, since they form a ‘force clamp’ that provides the primary resistance against rupturing of the protein by stretching from the N and C termini [28]. In Fig. 2b the residue-wise average main-chain forces within the beta force clamp are illustrated. Forces between neighboring, covalently-bonded residues are not shown, and will be discussed separately. There are five hydrogen bonds between these beta strands, formed by residues Gln2 and Glu64, Phe4 and Ser65, Phe4 and Leu67, Lys6 and Leu67, and Lys6 and Leu69; and it is evident that these pairs are precisely those for which the average pairwise force is repulsive (blue).

Apart from the hydrogen-bonded pairs, every other residue pair in beta strand pair 1/5 experiences an average attractive force (red lines in Fig. 2a); they are all pre-tensed. This gives the beta sheet an overall appearance reminiscent of a tensegrity structure, the mechanical stability of which is determined by a balance between tensed and compressed structural members [3]. The origin of the pre-compression of the hydrogen bonds can be understood via this tensegrity analogy: the ‘tensed’ attractive interactions between the two beta strands act to pull the strands closer together than they would otherwise like, compressing the hydrogen bonds until the tensile and compressive forces balance. The same pattern, of hydrogen bonds compressed by other attractive cross-strand interactions, also holds for the other beta strand pairs in the protein, both parallel and anti-parallel; see Fig. S1 for the force distributions in the anti-parallel beta strand pairs 1/2 and 3/5. The underlying atomic forces that give rise to the attractive and repulsive residue-residue forces are illustrated in Fig. S2.

To investigate how the combination of atomic forces gives rise to an effective force profile for each residue pair, we plot the distribution of residue-wise force versus separation of the C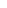 atoms. Fig. 2c shows the result of this procedure for the hydrogen-bonded residue pair Gln2-Glu64. Each point in the figure corresponds to a single frame of the trajectory. The scatter of the data points is large, due to fluctuations in the conformations and relative orientations of the two residues. The average fit (blue curve) represents an ‘effective’ pairwise force profile averaging over these fluctuations [29]. Around the mean separation, the effective force profile is approximately linear, and thus has the character of a compressed Hookean spring. But the curve is clearly non-linear at larger separations, approaching the rupture distance of the bond. The overall shape is reminiscent of a Morse-type potential traditionally used to approximate chemical bonds. Similar profiles are obtained for the other hydrogen bonds in the sheet.

Fig. 2d shows the effective force profile for one of the ‘tensed’ non-hydrogen-bonded pairs (Ile3-Ser65). The magnitude of the attractive force is found to reduce with separation. Such behaviour cannot be approximated by a physical Hookean spring, since the local effective spring constant is negative; it is instead more like a Morse-type potential where the interacting pair only samples the tail of the potential, never even approaching the equilibrium separation. Thus the analogy with macroscopic tensegrity structures is only superficial: it is not accurate to think of the tensed residue pairs as prestressed cables, which would exhibit Hookean behavior. Due to the partially non-Hookean springs in the network of ubiquitin, the prestress can be expected to have an impact on both the elastic behavior of the protein (if any) as well as the inelastic behavior including rupture.

Although the alpha helix does not play a direct role in determining the mechanical stability of the protein, it is interesting to look at the pattern of prestress in the helix and see whether pre-compression of hydrogen bonds is a general phenomenon or one restricted to beta sheets. Fig. S3 shows the main-chain-only residue-wise forces within the helix. Similar to the beta sheets, the helix exhibits a tensegrity-like pattern of balancing compressive and tensile forces. However, in this case the hydrogen bonded residue pairs are under *tension*, in contrast to the compressed beta-sheet hydrogen bonds. We conclude that pre-compression of hydrogen bonds is not a property intrinsic to all hydrogen bonds, but rather a context-dependent phenomenon: prestress in a given bond is determined by the interactions between other residues in its immediate neighborhood, and the local molecular geometry. This points to the fascinating possibility that the distribution of prestress in a protein can be *engineered* by intelligent modifications to the amino acid sequence, providing a new tool for designing proteins with made-to-order mechanical properties [30].

### Estimating the influence of hydrogen bond pre-compression on forced unfolding

**Figure 3:**
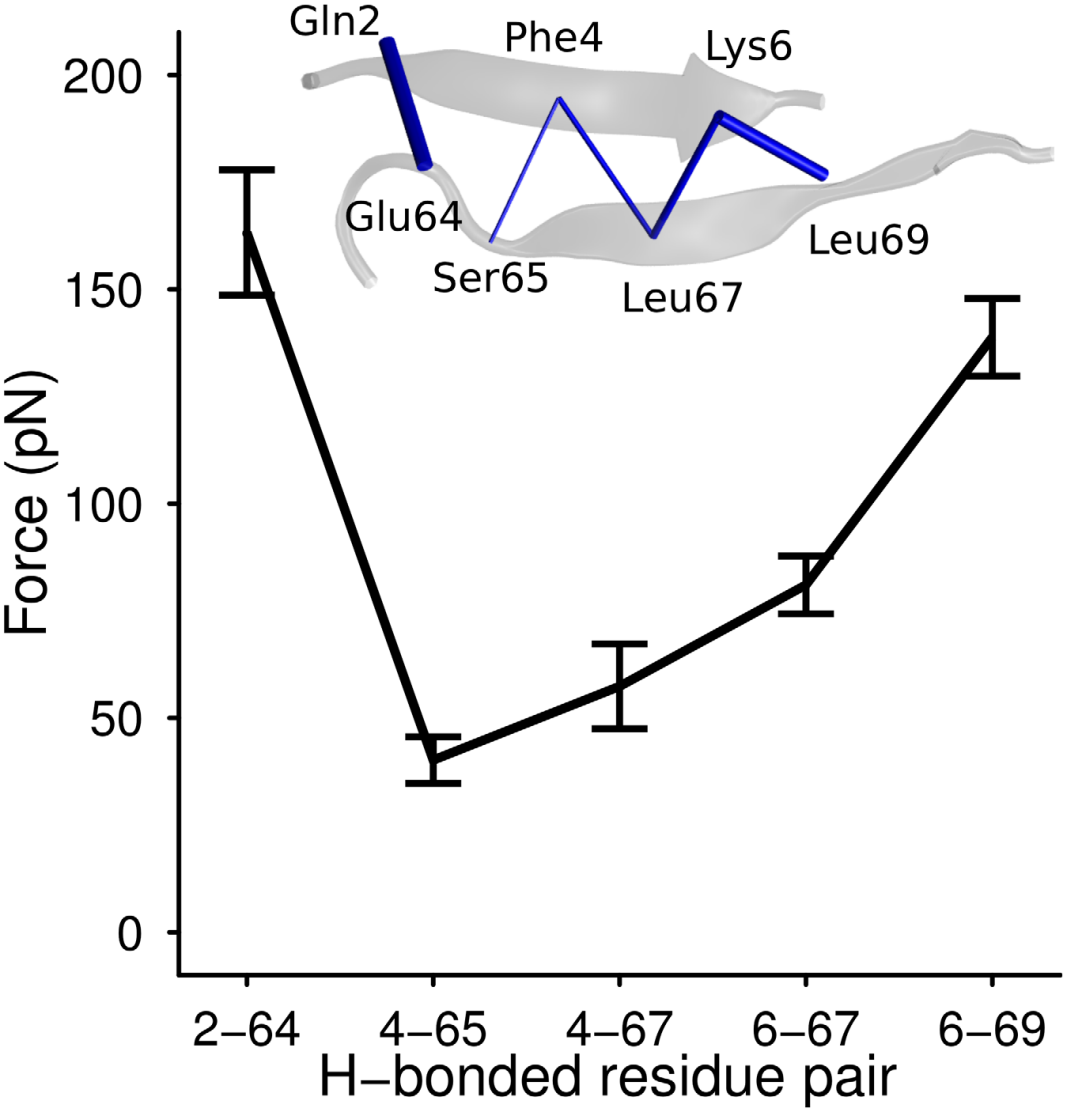
Average forces for each of the cross-strand hydrogen bonds in beta sheet 1–5. Error bars show the standard error in the mean.

Any applied external force must work against the inherent compression imposed by the protein onto the rupturing bonds. We propose that the hydrogen bond compression influences the unfolding force and pathway of the force clamp between beta strands 1 and 5. Fig. 3 is a plot of the average force for each of the five bonds in this clamp. Of the two bonds at the edge of the sheet, pair Gln2-Glu64 (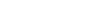) is significantly more compressed than Lys6-Leu69 (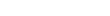). Arguing from Bell's theory of the rupture of individual bonds under force [31], it can be shown that pre-compression of a bond should increase its average lifetime. Based on the kinetic theory of thermally-activated rupture in metals [32], Bell wrote down the following expression for the lifetime 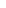 of a single bond subjected to an external force 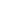:
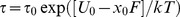
(1)where 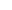 is the inverse of the atomic oscillation frequency (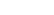 s), 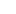 is the height of the energy barrier separating the bound and unbound states, and 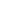 is a measure of the distance between the bound and transition states. If the bond is also subjected to a compressive ‘prestress’ force 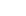, we then have
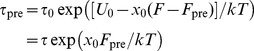
(2)Eq. 2 can be used to estimate the contribution of pre-compression to the lifetimes of the two end hydrogen bonds. For Gln2-Glu64, we have 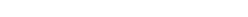 at room temperature, assuming 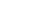
Å; the characteristic lifetime of the Gln2-Glu64 bond is enhanced by a factor of 400, with respect to a non-compressed hydrogen bond. The analogous calculation for the Lys6-Leu69 bond gives 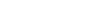, suggesting that hydrogen bond compression extends the lifetime of ubiquitin under a stretching force significantly, by approximately two orders of magnitude. We note that the elastic energy stored in such a prestressed hydrogen bond can be expected to be minor, as a force of 100 pN approximately corresponds to an energy of only approximately 1 J/mol.

The magnitude of compression of the hydrogen bonds is not uniform along the beta strand pair 1/5. We propose that differences in hydrogen bond compression influence the unfolding pathway for the beta force clamp. It is known from earlier MD simulation work [28], [33] that the Lys6-Leu69 hydrogen bond always ruptures first when the protein is unfolded by stretching the N- and C-termini. The stronger pre-compression of pair Gln2-Glu64 relative to pair Lys6-Leu69 should be a contributing factor in determining this unfolding pathway. The ratio of the lifetimes for the two edge hydrogen bonds is 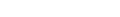. Thus, differences in pre-compression of hydrogen bonds of the magnitude we observe here are enough to more than double the relative lifetime of the more-compressed bond, all else being equal. This calculation is made under the assumption that the magnitude of pre-compression does not change as the protein is stretched, which is unlikely to be the case in reality; how the network of pre-tensile and pre-compressed forces evolves under an applied stretching force will be a topic for future study. Despite this simplification, our rough calculation serves to demonstrate that prestress is an important factor in determining a protein's mechanical stability, and should be taken into account along with other factors such as the orientation of the bonds relative to the pulling direction and the shielding from water by hydrophobic side-chains [33].

### Side-chain prestress

**Figure 4:**
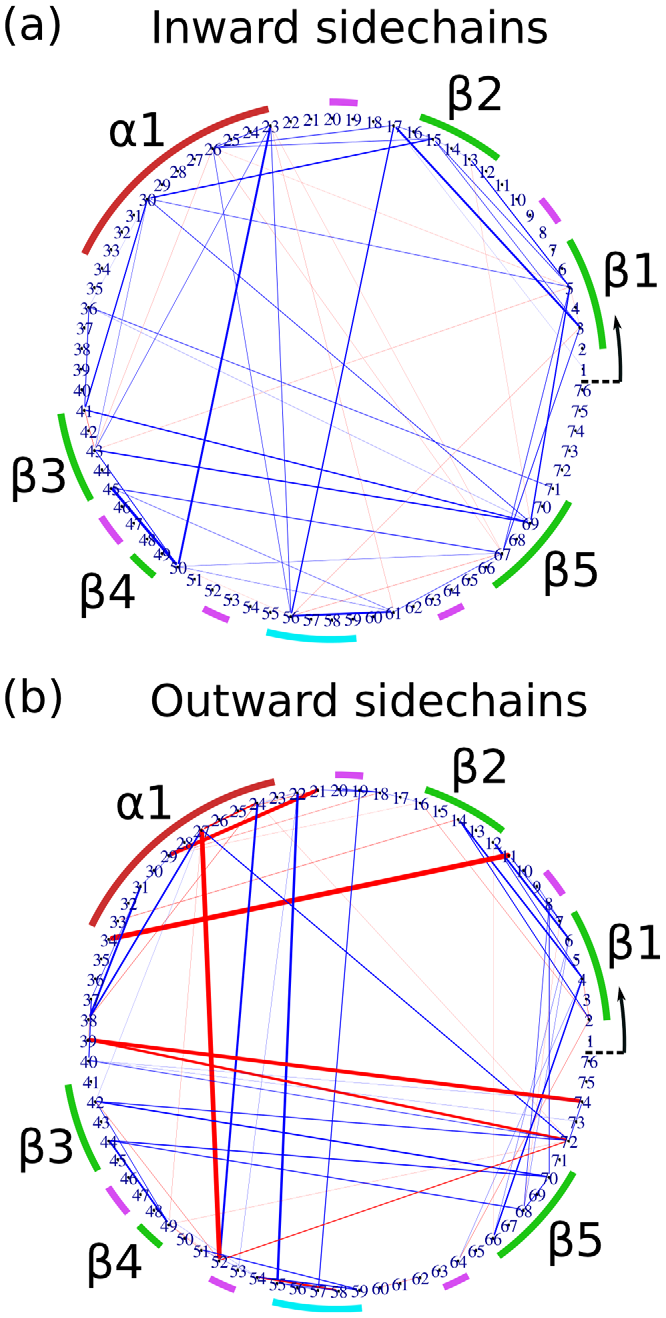
(a) and (b) Networks representing the inter-residue forces for ubiquitin, as for **Fig. 1c**, but accounting for side-chain atoms only; (a) inward-pointing side-chains, (b) outward-facing side-chains.

Apart from intra-main-chain interactions, we found that side-chain–side-chain interactions also exhibit prestress. For clarity, the inter-side-chain forces are separated into those for side-chains comprising the hydrophobic core of the protein (Fig. 4a) and for side-chains facing outwards into the solvent (Fig. 4b). The two are also shown together, projected on the protein structure, in Video S3. The inward-facing hydrophobic side-chains, with few exceptions, repel each other. None of their atoms are highly charged, and thus the inter-residue forces are dominated by steric repulsion. This is consistent with the hydrophobic core being compressed by tension in the ‘skin’ of the protein comprising the main-chain and outer side-chains, as well as by entropic forces related to the hydrophobic effect.

In contrast to the core side-chains, the forces between outward-facing side-chains are found to exhibit a mix of both compressive and tensile prestress. The strongest attractive forces (red in the figure) all correspond to salt bridges between charged side-chains (lysine and arginine are positively charged, aspartic acid and glutamic acid negatively charged). The pair with the highest tensile prestress is Lys11-Glu34, which comprises a salt bridge connecting the C-end of the alpha helix with the N-end of beta strand 2. This particular salt bridge has been shown experimentally to contribute significantly to the thermodynamic stability of ubiquitin [34]. Because of the relatively large distance between the two residues, their side-chains are forced to fully extend to satisfy the electrostatic attraction, giving rise to the observed prestress of the residue-residue force: the electrostatic attraction is counterbalanced by entropic stretching of the side-chains. It is generally true that the residue pairs with the strongest tensile prestress (eg. Asp21-Lys29, Lys27-Asp52, and Asp39-Arg74) are salt bridges between spatially separated residues. Conversely, salt bridges between nearby residues, such as Glu51-Arg54, can be satisfied without stretching the side-chains and accordingly the inter-residue forces show no significant prestress. We find evidence that some of the pre-tensed salt bridges generate significant torsion in the backbone, and this torque can be removed by mutating one of the salt bridge partners to ‘break’ the salt bridge (see Supplementary Text S1 for more details). Thus, side-chain prestress should be an important factor in stabilising the protein's native conformation.

Unlike the main-chain-only prestress network, for which each residue has significant interactions with at most two others, some nodes in the side-chain network are connected to as many as four or five others, widely separated in sequence-space. In the context of network theory, these residues may be thought of as ‘hubs’ of the network; perturbing these residues may be expected to lead to a wide-spread redistribution of force in the prestress network. In fact, simulations in which two of the most obvious hub residues, Asp52 and Arg72, were separately mutated to glycines exhibited no statistically significant changes to the prestress network beyond the local neighborhood of the mutated residue. This suggests that, at least with respect to perturbations of these specific residues, redundancy in the mechanical network imbues the pre-stress distribution with a certain amount of rigidity, and that intentional engineering of a protein's prestress network may require a more sophisticated mutation strategy beyond simply perturbing individual network hubs.

### Effective force profiles reveal complex side-chain dynamics

**Figure 5:**
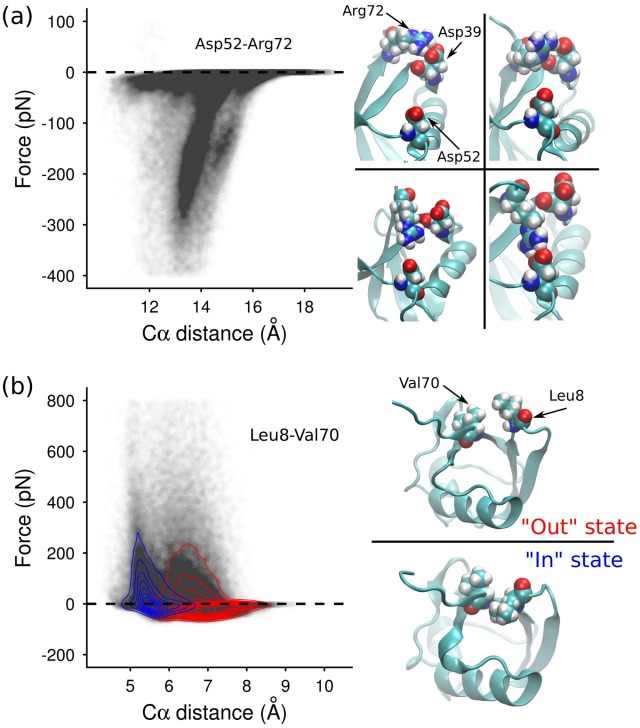
(a) Left: Effective force profile for the side-chains of Asp52 and Arg72. Right: Snapshots of various hydrogen binding states observed for the trio of Asp39, Asp52, and Arg72. Sometimes two of the amides in the Asp side-chain are simultaneously hydrogen bonded to the two oxygens in the Asp side-chain (upper left); sometimes an amide is bound to a side-chain oxygen, and another to the backbone carbonyl oxygen (upper right); sometimes only a single bond is formed, to the backbone oxygen (lower left); and sometimes the Asp39-Arg72 salt bridge breaks entirely, and Arg72 instead forms a short-lived salt bridge with Asp52, which is simultaneously bound to Lys27 (lower right). (b) As for (a), but for the residues Leu8 and Val70. In one state (the ‘out’ state), the side-chain of Leu8 is oriented outwards, above that of Val70. In the other (the ‘in’ state), the Leu8 side-chain is buried underneath Val70. Superimposed on the scatterplot are two density plots, which show the density of points belonging to each of the ‘in’ (red) and ‘out’ (blue) states. The classification of points into two states is described in Fig. S4.

The connections between the hub residues Lys27, Asp52 and Arg72 form a clear triangle in Fig. 4b, most notably featuring a strong tensile prestress between the salt-bridged residues Lys27 and Asp52. A clue to how the high connectivity of these hubs arises comes from the effective force profile for Asp52 and Arg72 (Fig. 5a). Unlike the main-chain hydrogen bond profiles, this distribution seems to show at least three separate overlapping force profiles. This suggests that the side-chains involved are visiting a number of distinct conformational states over the course of the simulation. We indeed find evidence of very complex dynamics for Arg72 and its neighbors, which alternately involves hydrogen bonds to Asp52 and other competing residues, involving their sidechains, backbone, or both (Fig. 5a, right). It is now possible to detect the dynamics of arginine side-chains from NMR [35], so it should be feasible to directly validate our predictions of Arg72's propensities for binding to its neighbors.

Such switching between discrete states is also observed for hydrophobic residues. Fig. 5b shows the effective force profile for the residue pair Leu8-Val70. These two residues are functionally important, since they comprise a hydrophobic binding patch that is crucial for the binding of Lys48-C-linked polyubiquitin to the proteasome [36]. The force-distance distribution seems to show two distinct force curves, one with an equilibrium separation around 5.5 Å, and another around 6.5 Å. The existence of two states is confirmed from examining representative states of the trajectories (Fig. 5b, right), as well as by analysing the distribution of the angle between the two side-chains as a function of residue-residue separation over the length of the simulation (Fig. S4). As for the Asp52-Arg72 pair discussed above, the overlapping effective force curves here reveal that these different orientational states for the side-chains correspond to different inter-residue mechanical coupling regimes. It is conceivable that the switching between these states has an influence on the local balance of tension and compression, and thus on the protein's mechanical stability. This degeneracy in mechanical stability may contribute to the signature of static disorder detected in ubiquitin's rupture kinetics as measured by recent AFM experiments [37], [38]. To what extent the local sidechain disorder influences the mechanical response might depend in nature on the type of polyubiquitin linkage, which is a topic for future research.

## Discussion

We have shown that forces in the native ensemble of ubiquitin, measured from all-atom MD simulations, generate a tensegrity-like pattern of prestress at the residue-residue level. This includes pre-compression of the hydrogen bonds connecting beta strands, and conversely pre-tensing of alpha helix hydrogen bonds. The differences between the pre-compression of individual beta strand hydrogen bonds are sufficient to significantly modify the kinetics of hydrogen bond breakage under force, and thus should be an important factor in determining the protein kinetic stability and unfolding pathway under mechanical perturbation. Salt bridges known to be important for ubiquitin's thermodynamic stability are found to be strongly pre-tensed, and the effective force profiles for side-chain–side-chain interactions reveal a connection between side-chain dynamics and inter-residue mechanical coupling. We propose that the observed dynamic equilibrium of multiple side-chain states contributes to the complex rupture kinetics observed in AFM experiments, since each discrete side-chain state corresponds to a different well in the rough global energy landscape. A correlation is found between tensed salt bridges and twisted peptide bonds in the protein backbone, which suggests that tension in stretched side-chains, transmitted as torque to the backbone, might play a role in determining the conformation of the protein's native state. Finally, we find the tensegrity network remarkably robust with regard to mutations at network hubs.

It remains to be shown whether the observations reported here apply generally to all proteins, or are specific to ubiquitin. A preliminary study of the titin immunoglobulin [I27] domain (PDB code 1WAA [23]) also found compression of hydrogen bonds in beta sheets, and tension in salt bridges, suggesting that these are general properties (Fig. S5). An early atomic force microscope study of the mechanical stability of I27 mutants [39] showed that the point mutations Val11Pro, Val13Pro and Val15Pro reduced the protein's rupture force, as expected due to proline's inability to form inter-strand hydrogen bonds; conversely, and unexpectedly, the mutant Tyr9Pro was found to be more stable than the wild type. Our I27 prestress network (Fig. S5) gives an intriguing clue as to the origin of this effect. Tyr9 is seen to be involved in a number of repulsive force pairs, with sequentially distant partners - not the case for Val11, Val13 and Val15. It may be that the mutation Tyr9Pro, by removing these frustrating repulsive forces, allows neighboring residues do adopt a more favorable conformation and thereby stabilise the protein. A detailed study of how such mechanically important point mutations involve changes to the prestress network will be a focus of future work, as will a survey of a wide range of protein structural types, necessary to better appreciate to what extent prestress is a ubiquitous aspect of protein structure. Futhermore, we have found that the prestress network is dynamic, due to the influence of side-chain dynamics on residue-residue forces, but more work needs to be done to quantify the relationship between applied force, side-chain states and protein function. Another question is whether the effective force profiles measured here can be used as a basis for prestressed coarse-grained protein models, and in what ways the predictions of such a model would differ from traditional elastic network models, which by definition lack any prestress. Our study opens the road to re-engineer molecular tensegrity structures, to eventually allow the rational tuning of mechanical or allosteric response.

## Materials and Methods

We used the Gromacs 4.0.5 package [40] to perform equilibrium all-atom simulations of ubiquitin, starting from the x-ray structure with PDB accession code 1UBI [41]. This structure is illustrated in Fig. 1a. The protein was solvated with TIP4P water [42] in a periodic cubic box of 6.5 nm per side. 16 pairs of sodium and calcium ions were added to give an effective salt concentration of 0.15 M. The OPLS all-atom forcefield [43] was chosen to describe interatomic energies. The system was subjected to a steepest-descent energy minimization, followed by a 1 ns solvent equilibration with position restraints on the heavy atoms of the protein. Then a further 1 ns equilibration run was performed with no position restraints. From the second half of the resulting trajectory, five snapshots were chosen to be the starting conformations for five independent production runs, each of which was carried out for 20 ns, giving a total of 100 ns of simulation time. All runs were performed in the NpT ensemble, with a Nosé-Hoover thermostat [44], [45] set to 300 K and Parrinello-Rahman barostat [46] at 1 atm, using a time-step of 2 fs. Electrostatic interactions were calculated using the particle mesh Ewald algorithm [47]. The same procedure was also carried out for the single-residue mutants Asp52Gly and Arg72Gly, initial structures of which were generated using PyMOL [48].

For each of the production runs, all pairwise atomic forces within the protein were output with a frequency of 1 ps using the modified FDA version of Gromacs 4.0.5 [23]. These pairwise atomic forces were then converted to residue-wise forces by summing in a vector-wise fashion, for each frame of the trajectory, all atomic forces between each pair of residues, and then projecting this total force on the vector connecting the C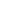 atoms of the two residues at that instant of the simulation. We note that due to the projection, any forces orthogonal to this connecting vectors, i.e. torques, are neglected. Their contribution to a protein's pre-stress will be subject of future investigations. The magnitudes of the residue-residue forces were then averaged for each residue pair over the full 100 ns of the simulation to give the average prestress distribution of the protein. Note that this procedure differs from earlier applications of FDA, in which residue-wise forces were calculated simply by summing the scalar magnitudes of the mean atomic pairwise forces. The protein-water and protein-ion forces were neglected. Effective force profiles for each pair of residues were obtained by selecting 10000 evenly-spaced frames from the total trajectory, and plotting the residue-wise force for each frame against the separation of the residues' C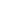 atoms. For studying the specific atomic contributions to inter-residue forces in more detail, the average atom-atom force distribution was also calculated, simply by averaging the total force between each pair of atoms in the protein over the 100 ns of simulation time. We refer to the network of forces in the protein also as ‘prestress’, in aid of establishing an analogy to previous work on the link between prestress and protein function and allostery, even though a normalization of forces by area has not been carried out, and ‘preforce’ would be the more accurate terminology. The standard error of the mean for time-averaged forces from the five independent trajectories was typically in the range of 10 pN, which is less than 10% of typical forces in hydrogen bonds, suggesting sufficient convergence. Protein visualisations were carried out with VMD [49] and PyMOL [48].

## Supporting Information

Figure S1 Average residue-residue forces in the antiparallel beta sheets 12 (left) and 15 right.
(PDF)

Figure S2 Average atomic forces for main-chain inter-residue interactions between Lys6 on beta strand 1, and Leu67, His68 and Leu69 on strand 5. Atom colors: cyan (carbon), blue (nitrogen), red (oxygen), white (hydrogen). Red (blue) lines represent attractive (repulsive) forces.
(PDF)

Figure S3 (a) Mean inter-residue forces (mainchain only) for the alpha helix. Vertices connecting residue pairs have the same meaning as in Fig. 1b. (b) The same forces plotted for i:i+2 (solid line), i:i+3 (dashed line) and i:i+4 (dotted line) pairs for each residue i in the helix. Note that i:i+4 pairs are hydrogen-bonded.
(PDF)

Figure S4For the residue pair Leu8/Val70, a scatterplot of the angle 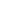 between the side-chains of the residues versus the C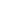-C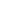 distance 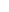. The directions of the side-chains are defined by the C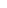-C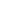 vector for Leu8 and the C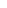-C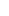 vector for Val70. Each dot in the scatterplot corresponds to a single frame taken from the 100 ns worth of MD production runs. There are two strong density peaks: one at around (6.8 Å, 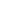), and another at (5.5 Å, 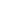), corresponding to the two distinct side-chain conformational states described in the main text. We arbitrarily define the boundary between the two states to be the straight line 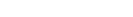. Dots above the line are assigned to the in state (blue) and dots below the line to the out state (red); this classification is then used in Fig. 5b for generating the two overlapping force-vs-distance density plots.(PDF)

Figure S5 (a) The network representing the inter-residue forces for the titin immunoglobulin domain, averaged over 50 ns of molecular dynamics simulations, superimposed on the 3D structure of the protein (PDB code 1WAA). The color and width of cylinders have the same meaning as Fig. 1b. (b) A circle graph representation of the prestress network in (a). The numbers around the circumference are residue indices. The green arcs show the locations of beta strands.
(PDF)

Figure S6(a) Average length and (b) average torsional angle (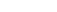) of the peptide bonds between neighboring residues i and i+1 along the protein backbone. In both cases, the grey area corresponds to the standard deviation. Standard error in the mean is smaller than the line width for (a), and 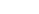 on average for (b). In (b), the red stars mark the two regions that deviate most strongly from the mean of around 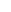.(PDF)

Figure S7

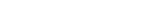
 for the Asp52Gly mutant, where 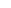 is the torsional angle of the peptide bond connecting neighboring residues i and i+1. The grey area shows the standard error in the mean. The dip around residues 51 and 52 corresponds to the eradication of the strong twist observed in wildtype ubiquitin (Fig. S6).(PDF)

Text S1 Backbone stress correlates with tensed salt bridges.
(PDF)

Video S1 Average inter-residue forces, measured from 100 ns of MD simulation, projected on the 3D structure of ubiquitin (PDB code 1UBI). Blue (red) cylinders represent repulsive (attractive) forces, and cylinder width is proportional to force magnitude, with the maximum force around 500 nN.
(MPG)

Video S2 As for Video S1, but forces are calculated using only main-chain atoms.
(MPG)

Video S3 As for Video S1, but forces are calculated using only side-chain atoms.
(MPG)
