# Supplementary figures and images for: Dynamic Prestress in a Globular Protein
Source: PLoS Comput Biol. 2012 May 10;8(5):e1002509. doi: 10.1371/journal.pcbi.1002509 (PMC3349725; doi:10.1371/journal.pcbi.1002509)

**$\beta$ -strand 2**

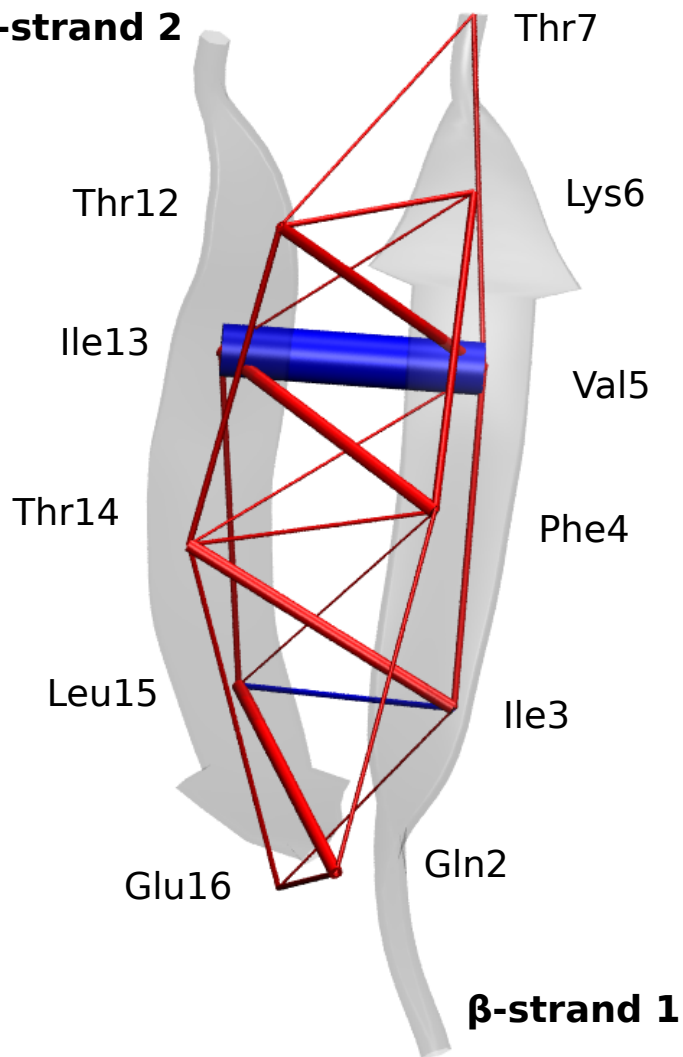

**$\beta$ -strand 3**

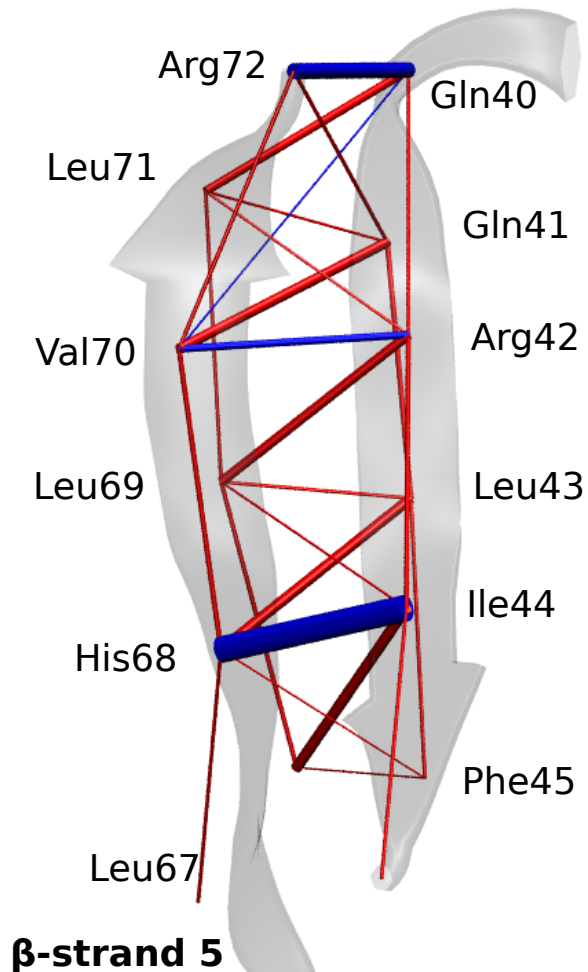

Supplement: Figure S1 — Average residue-residue forces in the antiparallel beta sheets 12 (left) and 15 right. (PDF) [file pcbi.1002509.s001.pdf]

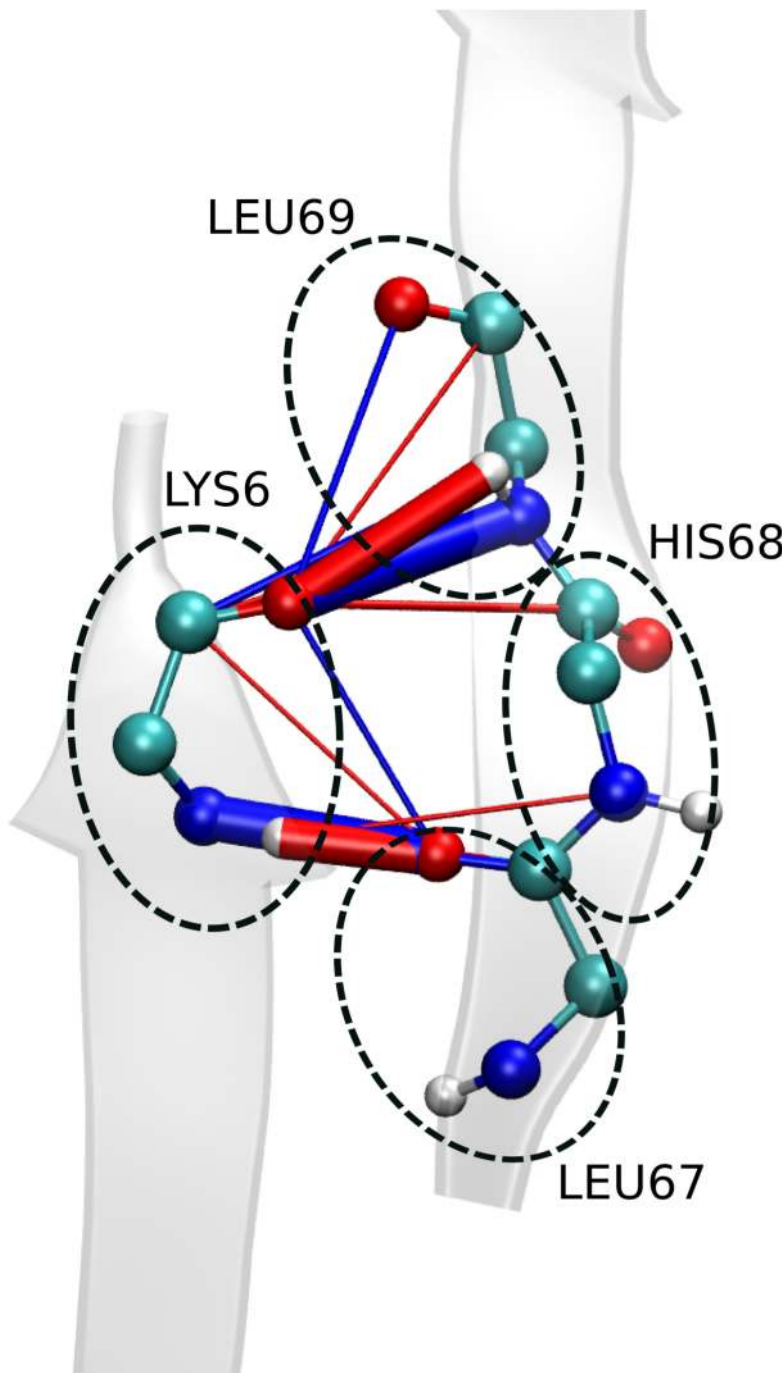

Supplement: Figure S2 — Average atomic forces for main-chain inter-residue interactions between Lys6 on beta strand 1, and Leu67, His68 and Leu69 on strand 5. Atom colors: cyan (carbon), blue (nitrogen), red (oxygen), white (hydrogen). Red (blue) lines represent attractive (repulsive) forces. (PDF) [file pcbi.1002509.s002.pdf]

(a)

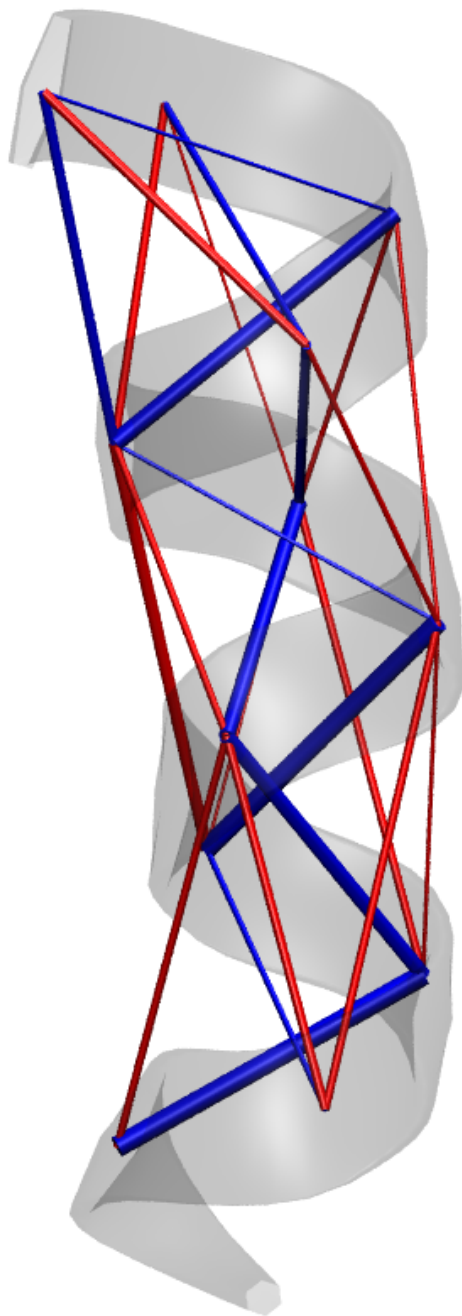

(b)

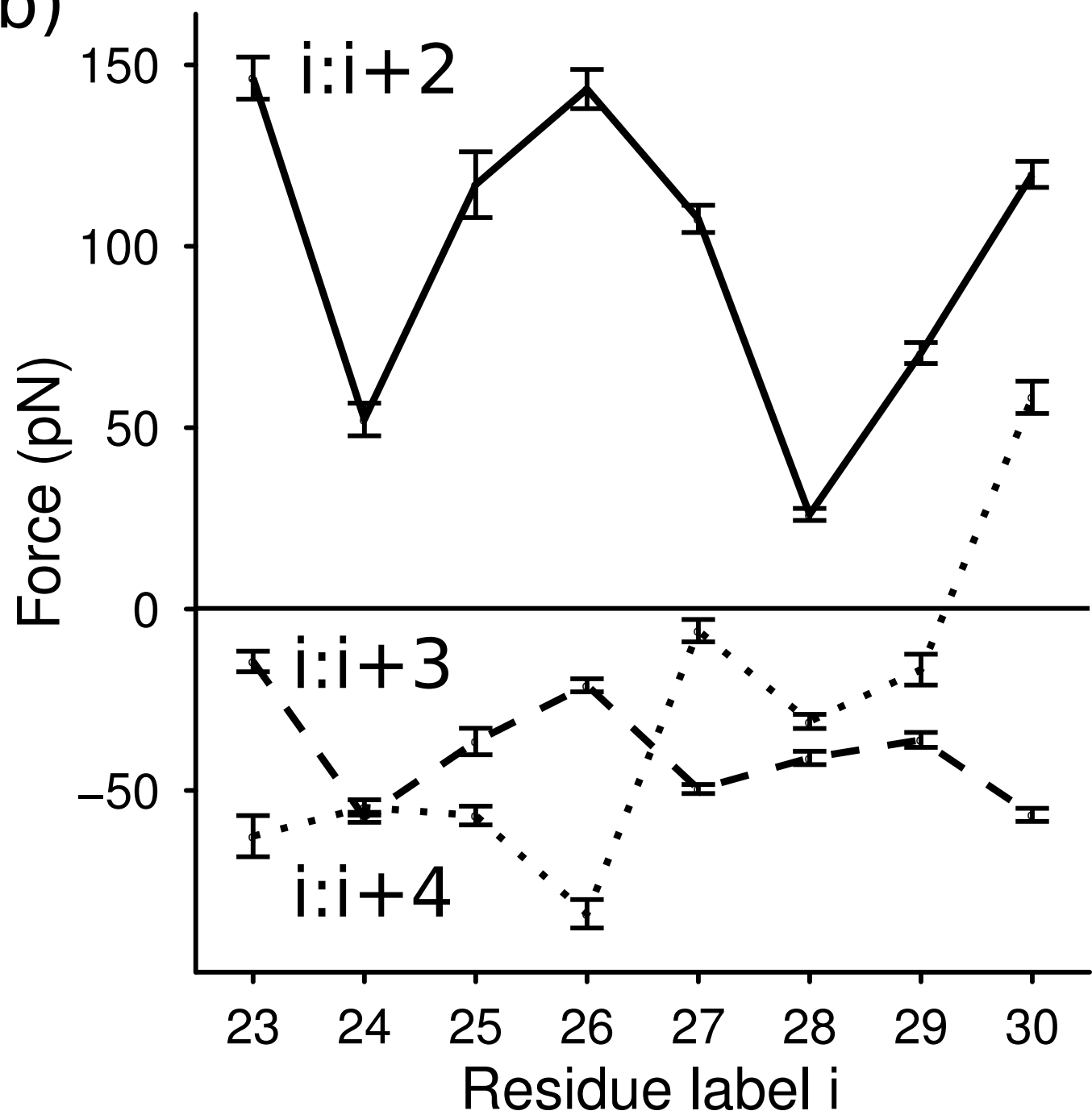

Supplement: Figure S3 — (a) Mean inter-residue forces (mainchain only) for the alpha helix. Vertices connecting residue pairs have the same meaning as in Fig. 1b. (b) The same forces plotted for i:i+2 (solid line), i:i+3 (dashed line) and i:i+4 (dotted line) pairs for each residue i in the helix. Note that i:i+4 pairs are hydrogen-bonded. (PDF) [file pcbi.1002509.s003.pdf]

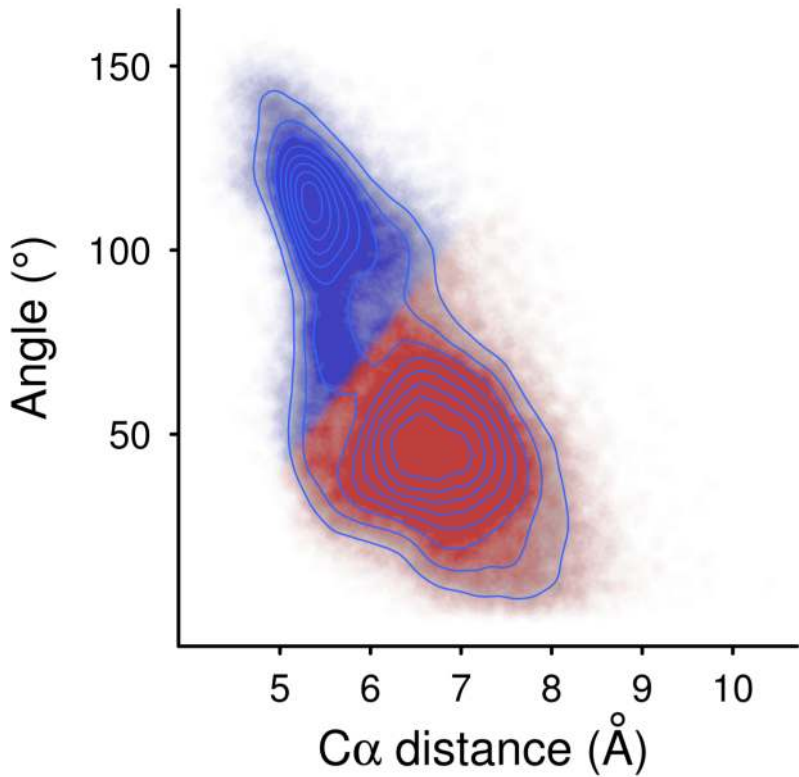

Supplement: Figure S4 — For the residue pair Leu8/Val70, a scatterplot of the angle between the side-chains of the residues versus the C-C distance . The directions of the side-chains are defined by the C-C vector for Leu8 and the C-C vector for Val70. Each dot in the scatterplot corresponds to a single frame taken from the 100 ns worth of MD production runs. There are two strong density peaks: one at around (6.8 Å, ), and another at (5.5 Å, ), corresponding to the two distinct side-chain conformational states described in the main text. We arbitrarily define the boundary between the two states to be the straight line . Dots above the line are assigned to the in state (blue) and dots below the line to the out state (red); this classification is then used in Fig. 5b for generating the two overlapping force-vs-distance density plots. (PDF) [file pcbi.1002509.s004.pdf]

(a)

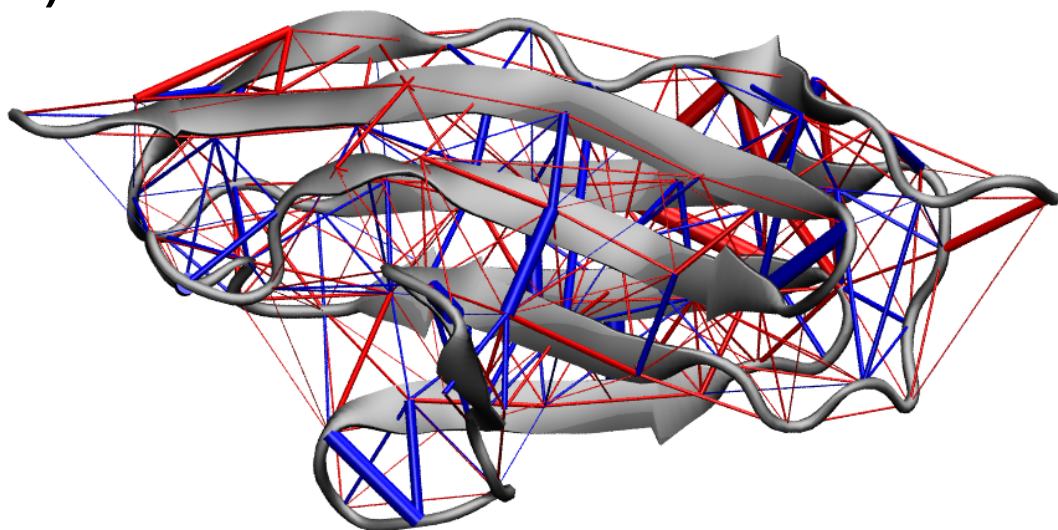

(b)

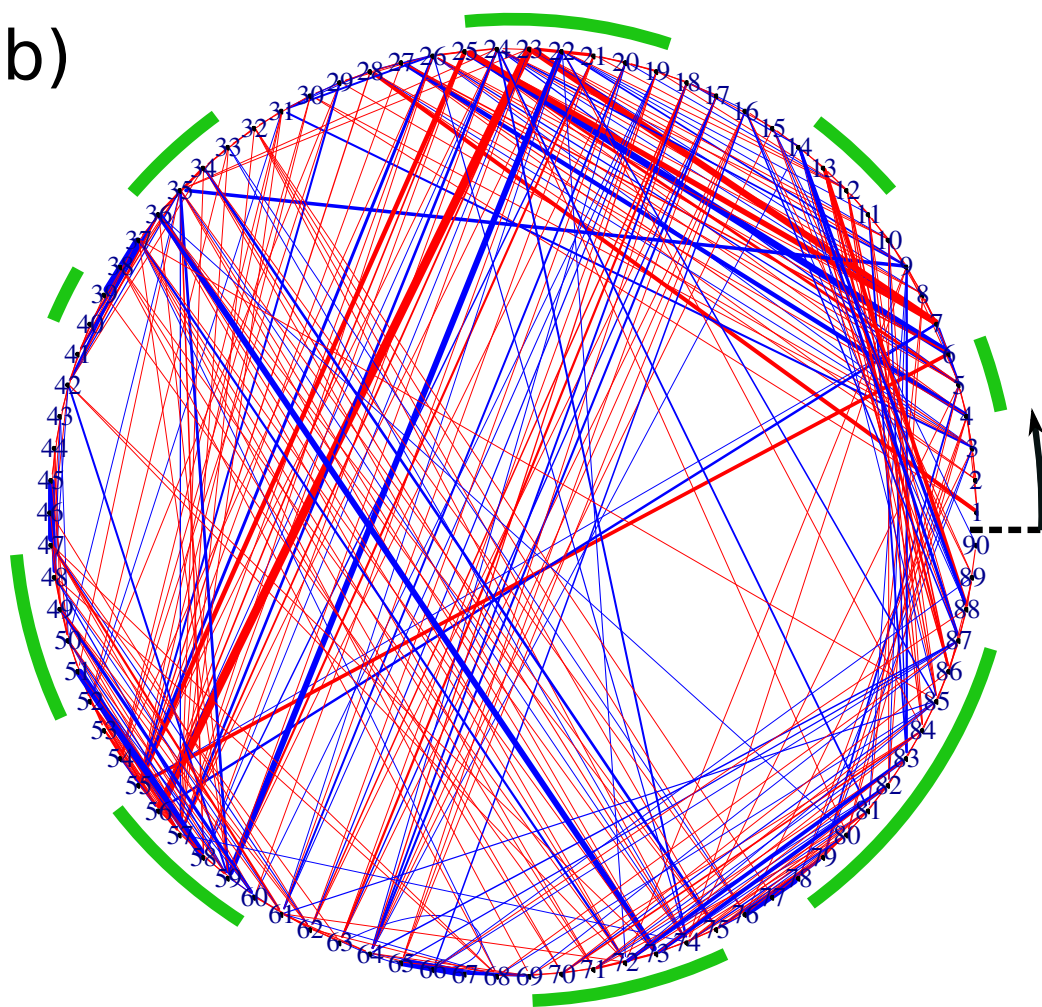

Supplement: Figure S5 — (a) The network representing the inter-residue forces for the titin immunoglobulin domain, averaged over 50 ns of molecular dynamics simulations, superimposed on the 3D structure of the protein (PDB code 1WAA). The color and width of cylinders have the same meaning as Fig. 1b. (b) A circle graph representation of the prestress network in (a). The numbers around the circumference are residue indices. The green arcs show the locations of beta strands. (PDF) [file pcbi.1002509.s005.pdf]

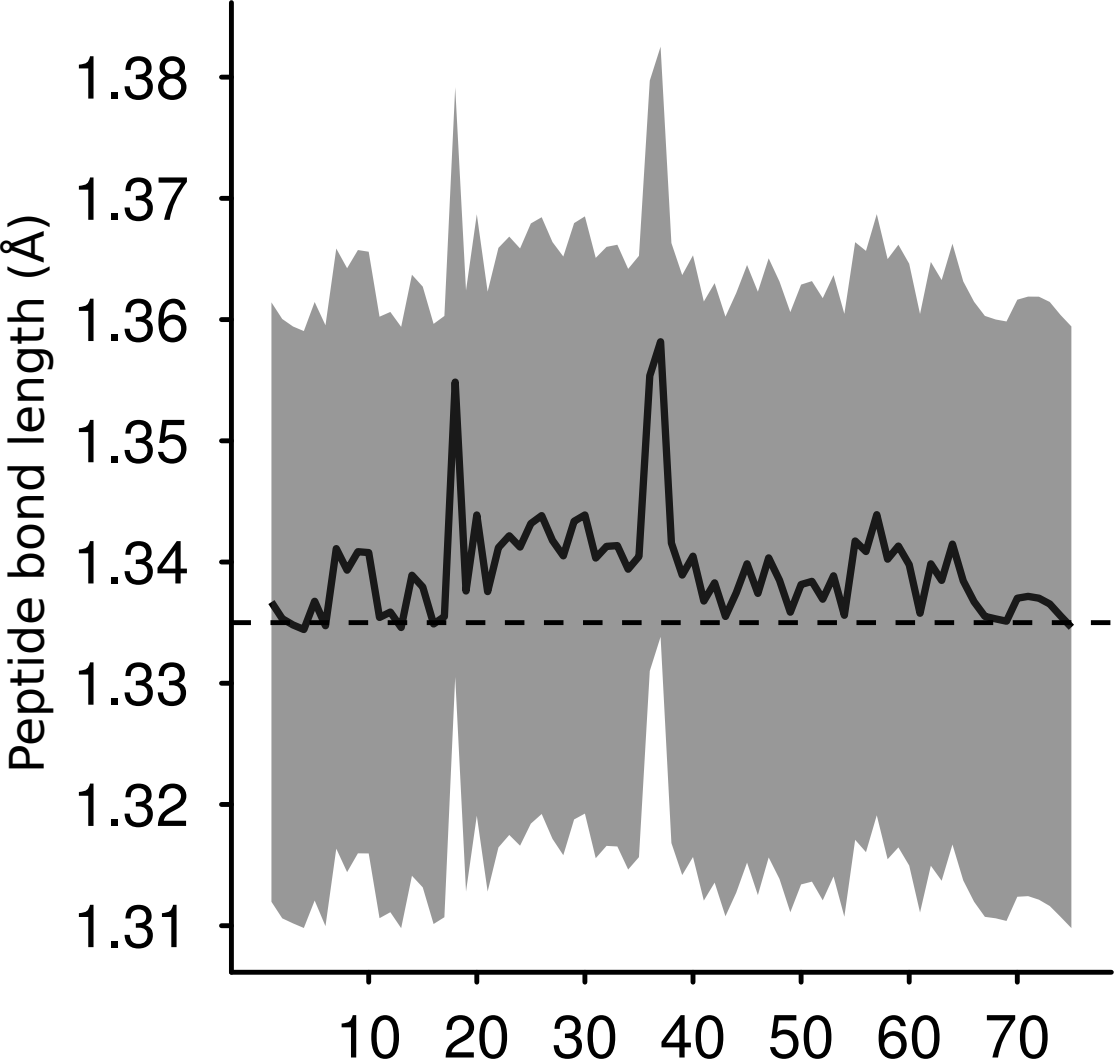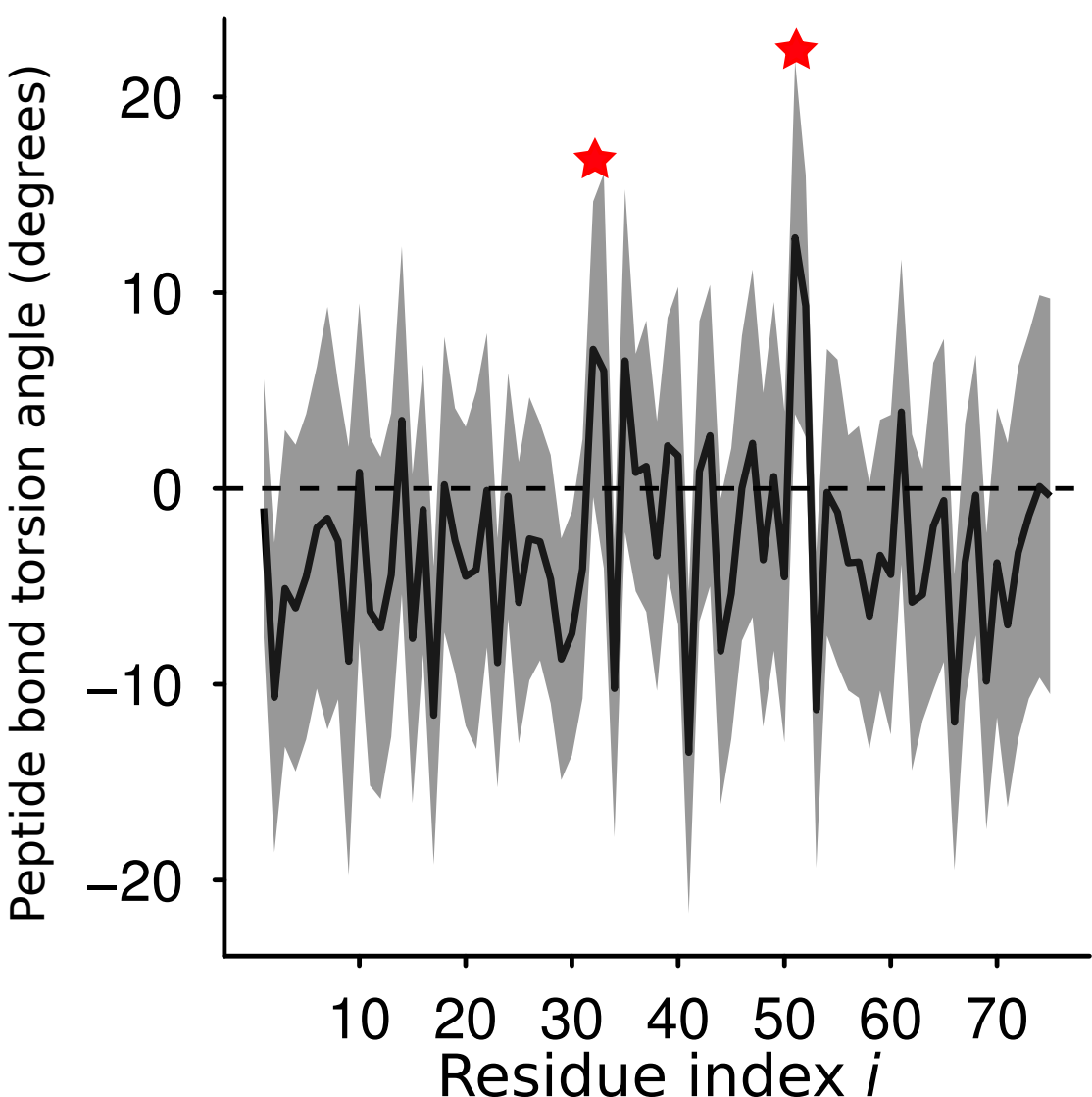

Supplement: Figure S6 — (a) Average length and (b) average torsional angle () of the peptide bonds between neighboring residues i and i+1 along the protein backbone. In both cases, the grey area corresponds to the standard deviation. Standard error in the mean is smaller than the line width for (a), and on average for (b). In (b), the red stars mark the two regions that deviate most strongly from the mean of around . (PDF) [file pcbi.1002509.s006.pdf]

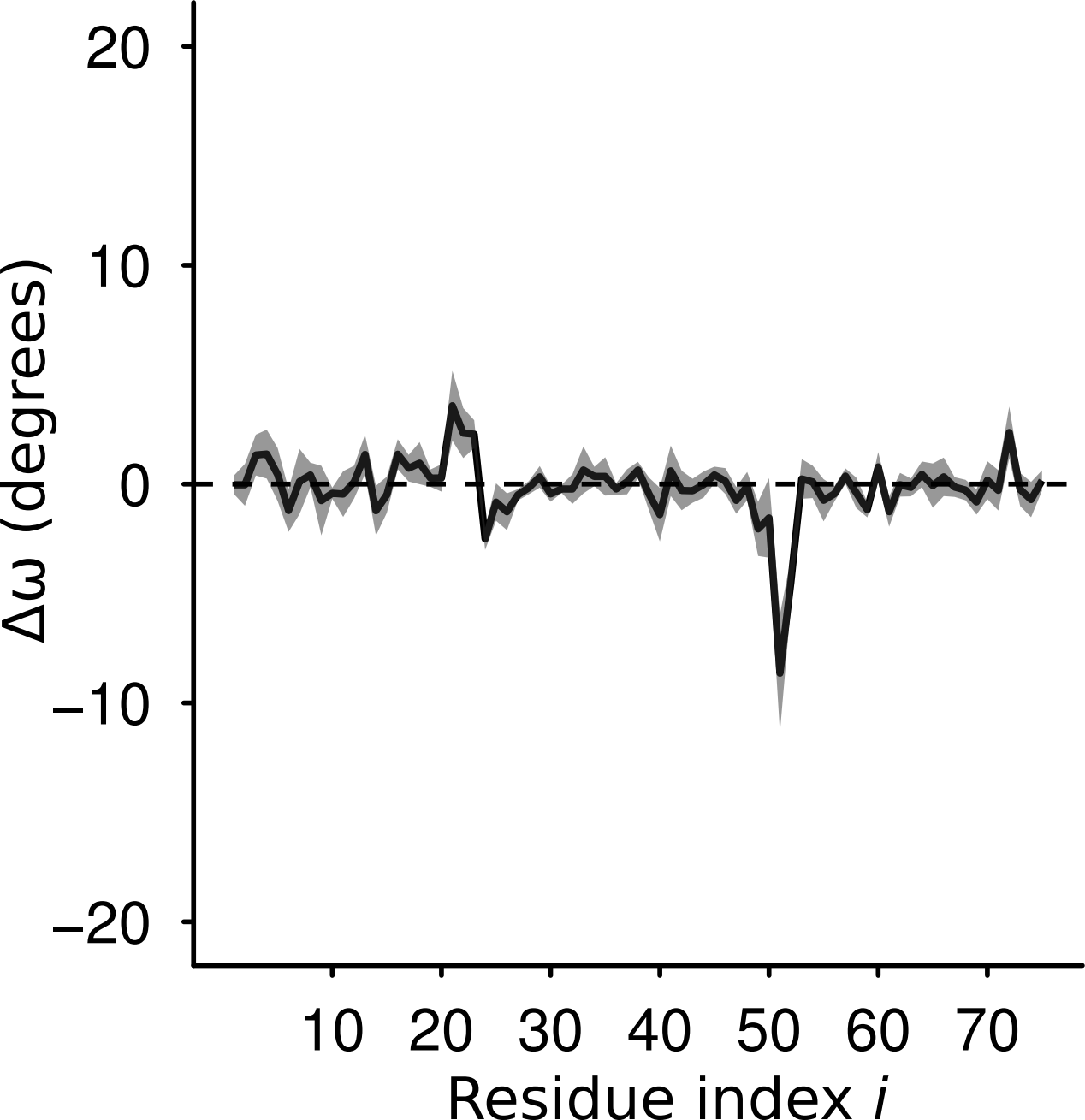

Supplement: Figure S7 — for the Asp52Gly mutant, where is the torsional angle of the peptide bond connecting neighboring residues i and i+1. The grey area shows the standard error in the mean. The dip around residues 51 and 52 corresponds to the eradication of the strong twist observed in wildtype ubiquitin (Fig. S6). (PDF) [file pcbi.1002509.s007.pdf]
