# Supplementary material for: Dynamic Prestress in a Globular Protein
Source: PLoS Comput Biol. 2012 May 10;8(5):e1002509. doi: 10.1371/journal.pcbi.1002509 (PMC3349725; doi:10.1371/journal.pcbi.1002509)
Supplement: Text S1 — Backbone stress correlates with tensed salt bridges. (PDF) [file pcbi.1002509.s008.pdf]

Dynamic Prestress in a Globular Protein

Scott A. Edwards, Johannes Wagner, and Frauke Gräter

Text S1

Backbone stress correlates with tensed salt bridges

The results discussed in the main text have all neglected interactions between covalently bonded residue pairs - i.e. those that neighbor each other along the peptide chain. We inferred the prestress present in covalent bonding interactions, some of which are not pair-wise but of multi-body nature, by directly measuring how much the average lengths of the peptide bonds and their associated angles deviate from their equilibrium values.

Fig. S6a shows the length of each inter-residue peptide (C-N) bond, averaged over the entire 100 ns-worth of trajectories. The equilibrium length in the OPLS forcefield is 1.335 Å. There are two distinct peaks, around residues 18-19 and 36-38, where the bond length jumps to 1.35 Å and 1.38 Å respectively; this is due solely to the proline residues at positions 19, 37 and 38, the strained cyclic side-chains of which are bonded to the backbone nitrogen and thereby stretch the peptide bond with the preceding residue. Apart from the prolines, there is also a correlation between stretch and secondary structure: a significant stretch (to 1.34 Å) is seen for helical and loop regions, while little stretch is seen in the beta strands.

The dihedral angle ω is generally assumed to be planar ($\omega = 180^\circ$), due to the partial double-bond character of the peptide bond, and as such the peptide bond torsional potential is made relatively stiff in MD forcefields. Despite this, it is clear from Fig. S6b that the mean value of ($180^\circ - \omega$) is in fact not zero, but around -5°, which is consistent with the known right-handed twist of globular proteins. Two peaks in the figure that deviate most strongly from the mean are marked with stars. These strongly twisted peptide bonds are both located very near two of the most strongly pre-tensed salt bridges: Lys11-Glu34 and Lys27-Asp52. This suggests that the stressed side-chains may be applying a significant torque to the backbone. It follows that if these salt bridges are 'broken', for example by binding to a ligand or another protein, the release of the tension could drive allosteric changes in the protein conformation. We tested this hypothesis by carrying out MD simulations of the ubiquitin mutant Asp52Gly, in which the Lys27-Asp52 is effectively broken. The difference in average ω values between the Asp52Gly mutant and wild-type ubiquitin are shown in Fig. S7: the breaking of the Lys27-Asp52 salt bridge is indeed found to eradicate the strong twist observed near Asp52 in the wild-type simulation.
